# Supplementary material for: An open-source framework for large-scale, flexible evaluation of biomedical text mining systems
Source: J Biomed Discov Collab. 2008 Jan 29;3:1. doi: 10.1186/1747-5333-3-1 (PMC2276192; doi:10.1186/1747-5333-3-1)
Supplement: Additional file 1 — Evaluation platform source code. The additional file contains the software evaluation framework discussed in this paper. The most current release can be downloaded from SourceForge.net [1]. See the accompanying README file for instructions on installation and use. Also included in the distribution is a collection of UIMA wrappers for some commonly used BioNLP tools and annotated corpora. [file 1747-5333-3-1-S1.zip › bionlp_uima_repository-1.0.1/docs/api/src-html/edu/uchsc/ccp/uima/util/opendmap/AnnotationString.html]

```
001    /**
002     * The OpenDMAP for Protege Project
003     * March 2006
004     */
005    package edu.uchsc.ccp.uima.util.opendmap;
006    
007    import java.util.ArrayList;
008    
009    import org.apache.uima.jcas.cas.FSArray;
010    
011    import edu.uchsc.ccp.uima.annotation.CCPSpan;
012    import edu.uchsc.ccp.uima.annotation.CCPTextAnnotation;
013    import edu.uchsc.ccp.uima.mention.CCPClassMention;
014    import edu.uchsc.ccp.uima.mention.CCPComplexSlotMention;
015    
016    /**
017     * This class generates a string describing a JCas CCPTextAnnotation in a standard
018     * format that can be used for generating and executing regression tests.
019     * 
020     * @author R. James Firby
021     */
022    public class AnnotationString {
023            
024            // Cached version of the output string once created.
025            private String myString = null;
026            
027            /**
028             * Create an object that will print as a standard format string for a JCas annotation.
029             * 
030             * @param annotation The annotation to describe in the string.
031             * @param slots The annotation slots to include.  Slots with names not in this list are excluded.
032             * @param docId The ID of the document associated with this annotation
033             * @param docText The document text associated with this annotatation
034             */
035            public AnnotationString(CCPTextAnnotation annotation, String slots[], String docId, String docText) {
036                    myString = create(annotation, slots, docId, docText);
037            }
038            
039            /**
040             * Return the standard format string for this annotation.
041             */
042            public String toString() {
043                    if (myString != null) return myString;
044                    return "";
045            }
046            
047            /**
048             * Create a new standard format string for a JCas annotation.
049             * 
050             * @param annotation The annotation to describe in the string.
051             * @param slots The annotation slots to include.  Slots with names not in this list are excluded.
052             * @param docId The ID of the document associated with this annotation
053             * @param docText The document text associated with this annotatation
054             */
055            public static String create(CCPTextAnnotation annotation, String slotNames[], String docId, String docText) {
056                    // Pull the semantic mention out of the annotation
057                    CCPClassMention mention = annotation.getClassMention();
058                    String base = toTestString(getCCPClassMentionBaseSpan(mention, docText), mention.getMentionName());
059                    // Begin creating the output string
060                    StringBuffer sb = new StringBuffer();
061                    if (docId != null) {
062                            sb.append(docId);
063                            sb.append("|");
064                    }
065                    sb.append(toSafeString(base));
066                    // Extract the slot fillers we care about
067                    if ((slotNames != null) && (slotNames.length > 0)) {
068                            String slotStrings[] = new String[slotNames.length];
069                            // Look at each slot
070                            FSArray slots = mention.getSlotMentions();
071                            if (slots != null) {
072                                    for (int i=0; i<slots.size(); i++) {
073                                            Object jcasSlotMention = slots.get(i);
074                                            CCPComplexSlotMention slot = null;
075                                            if (jcasSlotMention instanceof CCPComplexSlotMention) slot = (CCPComplexSlotMention) jcasSlotMention;
076                                            if (slot != null) {
077                                                    String slotValue = null;
078                                                    FSArray slotClassMentions = slot.getClassMentions();
079                                                    for (int j=0; j<slotClassMentions.size(); j++) {
080                                                            Object slotClassMention = slotClassMentions.get(j);
081                                                            CCPClassMention classMention = null;
082                                                            if (slotClassMention instanceof CCPClassMention) classMention = (CCPClassMention) slotClassMention;
083                                                            if (classMention != null) {
084                                                                    //System.out.println("Mention: " + classMention.getMentionName());
085                                                                    TextSpan filler = getCCPClassMentionSpan(classMention, docText);
086                                                                    if (slotValue == null) {
087                                                                            slotValue = toTestString(filler, classMention.getMentionName());
088                                                                    } else {
089                                                                            slotValue = slotValue + ";" + toTestString(filler, classMention.getMentionName());
090                                                                    }
091                                                            }
092                                                    }
093                                                    if (slotValue != null) {
094                                                            String slotName = slot.getMentionName();
095                                                            if (slotName != null) {
096                                                                    insertSlotString(slotNames, slotName, slotStrings, slotValue);
097                                                            }
098                                                    }
099                                            }
100                                    }
101                            }
102                            // Now add the slots of interest in order
103                            for (int i=0; i<slotStrings.length; i++) {
104                                    sb.append("|");
105                                    sb.append(toSafeString(slotStrings[i]));                                
106                            }
107                    }
108                    // Done
109                    return sb.toString();
110            }
111            
112            /**
113             * Place the value of slot into the corresponding array position as its slot name.
114             * 
115             * @param slotNames The names of the slots of interest
116             * @param slotName The name for the slot being processed
117             * @param slotStrings The values of the slots of interest
118             * @param slotValue The value of the slot being processed
119             */
120            private static void insertSlotString(String slotNames[], String slotName, String slotStrings[], String slotValue) {
121                    for (int i=0; i<slotNames.length; i++) {
122                            if (slotName.equalsIgnoreCase(slotNames[i])) {
123                                    slotStrings[i] = slotValue;
124                            }
125                    }
126            }
127            
128            /**
129             * Generate a standard format string component for a text span from a particular source.
130             * 
131             * @param span The text span including the text and the start and end character positions
132             * @param source The source of this text span, often an external UIMA annotator
133             * @return The formatted string component
134             */
135            private static String toTestString(TextSpan span, String source) {
136                    if (span == null) return "";
137                    if (source == null)
138                            return span.text + "[" + span.start + ".." + span.end + "]";
139                    return span.text + "[" + span.start + ".." + span.end + "]{" + source + "}";
140            }
141            
142            private static String toSafeString(String string) {
143                    if (string == null) return "";
144                    return string;
145            }
146    
147      /**
148       * Create a string that includes all of the spans within this mention.
149       * This includes recursively pulling out slot spans as well.
150       * 
151       * @param mention The mention to process
152       * @param data The original document text
153             * @return A string summarizing all the text spans included in this mention
154       */
155            private static TextSpan getCCPClassMentionSpan(CCPClassMention mention, String data) {
156                    // Create text spans for the base annotation and all the slots
157                    ArrayList<TextSpan> textSpans = new ArrayList<TextSpan>();
158                    addClassMentionSpans(textSpans, mention, data);
159                    // Assemble the sorted spans into a single string
160                    TextSpan result = appendSpans(textSpans);
161                    //result.source = mention.getMentionName();
162                    return result;
163            }
164            
165            /**
166             * Create the 'base' span for this mention.  That is the spans not included in a slot.
167             * @param mention The mention to process
168             * @param data The original document text
169             * @return A string capturing the base spans for this mention
170             */
171            private static TextSpan getCCPClassMentionBaseSpan(CCPClassMention mention, String data) {
172                    // Create text spans for the base annotation only
173                    ArrayList<TextSpan> textSpans = new ArrayList<TextSpan>();
174                    addClassMentionBaseSpans(textSpans, mention, data);
175                    // Assemble the sorted spans into a single string
176                    return appendSpans(textSpans);
177            }
178            
179            /**
180             * Add all of the text spans that take part in a mention into a sorted list of text spans.
181             * 
182             * @param textSpans The sorted list of text spans
183             * @param mention The mention from which spans are to be extracted
184             * @param data The original document text
185             * @return The sorted list of text spans
186             */
187            private static ArrayList<TextSpan> addClassMentionSpans(ArrayList<TextSpan> textSpans, CCPClassMention mention, String data) {
188                    // Get the base annotation for this mention (it should point to a CCPTextAnnotation)
189                    addClassMentionBaseSpans(textSpans, mention, data);
190                    // Now sort in all the spans from the mention slots
191                    FSArray slots = mention.getSlotMentions();
192                    if (slots != null) {
193                            for (int i=0; i<slots.size(); i++) {
194                                    Object jcasSlotMention = slots.get(i);
195                                    CCPComplexSlotMention slot = null;
196                                    if (jcasSlotMention instanceof CCPComplexSlotMention) slot = (CCPComplexSlotMention) jcasSlotMention;
197                                    if (slot != null) {
198                                            FSArray slotClassMentions = slot.getClassMentions();
199                                            for (int j=0; j<slotClassMentions.size(); j++) {
200                                                    Object slotClassMention = slotClassMentions.get(j);
201                                                    CCPClassMention classMention = null;
202                                                    if (slotClassMention instanceof CCPClassMention) classMention = (CCPClassMention) slotClassMention;
203                                                    if (classMention != null) {
204                                                            addClassMentionSpans(textSpans, classMention, data);
205                                                    }
206                                            }
207                                    }
208                            }
209                    }
210                    // Done
211                    return textSpans;
212            }
213    
214            /**
215             * Add all of the text spans that are part of a base mention into a sorted list of text spans.
216             * 
217             * @param textSpans The sorted list of text spans
218             * @param mention The mention from which spans are to be extracted
219             * @param data The original document text
220             * @return The sorted list of text spans
221             */
222            private static ArrayList<TextSpan> addClassMentionBaseSpans(ArrayList<TextSpan> textSpans, CCPClassMention mention, String data) {
223                    // Get the base annotation for this mention (it should point to a CCPTextAnnotation)
224                    FSArray annotations = mention.getCcpTextAnnotations();
225                    if ((annotations == null) || (annotations.size() <= 0)) return textSpans;
226                    Object jcasAnnotation = annotations.get(0);
227                    CCPTextAnnotation annotation = null;
228                    if (jcasAnnotation instanceof CCPTextAnnotation) annotation = (CCPTextAnnotation) jcasAnnotation;
229                    if (annotation == null) return textSpans;
230                    // Get all the spans from the base annotation
231                    FSArray spans = annotation.getSpans();
232                    if (spans == null) {
233                            sortIn(textSpans, new TextSpan(annotation.getStart(), annotation.getEnd(), data.substring(annotation.getStart(), annotation.getEnd())));
234                    } else {
235                            for (int i=0; i<spans.size(); i++) {
236                                    Object jcasSpan = spans.get(i);
237                                    CCPSpan span = null;
238                                    if (jcasSpan instanceof CCPSpan) span = (CCPSpan) jcasSpan;
239                                    if (span != null)
240                                            sortIn(textSpans, new TextSpan(span.getSpanStart(), span.getSpanEnd(), data.substring(span.getSpanStart(), span.getSpanEnd())));
241                            }
242                    }
243                    // Done
244                    return textSpans;
245            }
246            
247            /**
248             * Append a list of spans together to create a single span.
249             * 
250             * @param textSpans The spans to append
251             * @return The resulting single span
252             */
253            private static TextSpan appendSpans(ArrayList<TextSpan> textSpans) {
254                    StringBuffer sb = new StringBuffer();
255                    TextSpan previous = null;
256                    for (TextSpan span: textSpans) {
257                            if (previous == null) {
258                                    sb.append(span.text);
259                            } else if ((span.start - previous.end) <= 1) {
260                                    sb.append(" ");
261                                    sb.append(span.text);
262                            } else {
263                                    sb.append(" ... ");
264                                    sb.append(span.text);
265                            }
266                            previous = span;
267                    }
268                    if (textSpans.size() == 0) {
269                            return new TextSpan(0, 0, "");
270                    } else {
271                            return new TextSpan(textSpans.get(0).start, textSpans.get(textSpans.size()-1).end, sb.toString());
272                    }
273            }
274    
275            /**
276             * Sort a new text span into a list of text spans.
277             * 
278             * @param spans The sorted list of spans
279             * @param span The new span to add
280             * @return The sorted list of spans
281             */
282            private static ArrayList<TextSpan> sortIn(ArrayList<TextSpan> spans, TextSpan span) {
283                    if (spans.isEmpty()) {
284                            spans.add(span);
285                    } else {
286                            boolean found = false;
287                            for (int i=0; i<spans.size(); i++) {
288                                    if (span.end <= spans.get(i).start) {
289                                            spans.add(i, span);
290                                            found = true;
291                                            break;
292                                    }
293                            }
294                            if (!found) spans.add(span);
295                    }
296                    return spans;
297            }
298    
299    }
300    /**
301     * A local class to hold a span of text along with its document start and end position.
302     * 
303     * @author Jim Firby
304     */
305    class TextSpan {
306            
307            int start = 0;
308            int end = 0;
309            String text = null;
310            //String source = null;
311            
312            TextSpan(int start, int end, String text) { //String source) {
313                    this.start = start;
314                    this.end = end;
315                    this.text = text;
316                    //this.source = source;
317            }
318            
319            //TextSpan(int start, int end, String text) {
320            //      this(start, end, text, null);
321            //}
322    
323    }
324
```
